# Transient early life growth hormone exposure permanently alters brain, muscle, liver, macrophage, and adipocyte status in long‐lived Ames dwarf mice

**DOI:** 10.1096/fj.202200143R

**Published:** 2022-06-15

**Authors:** Xinna Li, Madaline McPherson, Mary Hager, Yimin Fang, Andrzej Bartke, Richard A. Miller

**Affiliations:** ^1^ Department of Pathology University of Michigan School of Medicine Ann Arbor Michigan USA; ^2^ College of Literature, Sciences, & the Arts University of Michigan Ann Arbor Michigan USA; ^3^ Department of Internal Medicine Southern Illinois University School of Medicine Springfield Illinois USA; ^4^ University of Michigan Geriatrics Center Ann Arbor Michigan USA

**Keywords:** Ames dwarf (DF) mice, fibronectin type III domain‐containing protein 5 (FNDC5), glycosylphosphatidylinositol specific phospholipase D1 (GPLD1), growth hormone, uncoupling protein 1 (UCP1)

## Abstract

The exceptional longevity of Ames dwarf (DF) mice can be abrogated by a brief course of growth hormone (GH) injections started at 2 weeks of age. This transient GH exposure also prevents the increase in cellular stress resistance and decline in hypothalamic inflammation characteristic of DF mice. Here, we show that transient early‐life GH treatment leads to permanent alteration of pertinent changes in adipocytes, fat‐associated macrophages, liver, muscle, and brain that are seen in DF mice. Ames DF mice, like Snell dwarf and GHRKO mice, show elevation of glycosylphosphatidylinositol specific phospholipase D1 in liver, neurogenesis in brain as indicated by BDNF and DCX proteins, muscle production of fibronectin type III domain‐containing protein 5 (a precursor of irisin), uncoupling protein 1 as an index of thermogenic capacity in brown and white fat, and increase in fat‐associated anti‐inflammatory macrophages. In each case, transient exposure to GH early in life reverts the DF mice to the levels of each protein seen in littermate control animals, in animals evaluated at 15–18 months of age. Thus, many of the traits seen in long‐lived mutant mice, pertinent to age‐related changes in inflammation, neurogenesis, and metabolic control, are permanently set by early‐life GH levels.

**Abbreviations**
ADRβ3: β‐3‐adrenergic receptor
ARG1: arginase 1
ATM: adipose tissue macrophages
BAT: brown adipose tissue
BDNF: brain‐derived neurotrophic factor
CIT: cap‐independent translation
DCX: doublecortin
DF: ames dwarf
DIO2: iodothyronine deiodinase 2
FNDC5: fibronectin type III domain‐containing protein 5
GH: growth hormone
GHR: growth hormone receptor
GHRH: growth hormone‐releasing hormone
GHRHR: growth hormone‐releasing hormone receptor
GHRKO: growth hormone receptor knockout
GPI: glycosylphosphatidylinositol
GPLD1: glycosylphosphatidylinositol specific phospholipase D1
IGF1: insulin like growth factor 1
ING: inguinal adipose tissue
iNOS: inducible nitric oxide synthase
NFkB: nuclear factor kappa B subunit 1
PE: perigonadal adipose tissue
PPARγ: peroxisome proliferator‐activated receptor gamma
UCP1: uncoupling protein 1
WAT: white adipose tissue

## 1 INTRODUCTION

Changes in the pituitary that reduce production of growth hormone (GH), thyroid stimulating hormone, and prolactin lead to a dramatic increase in mean and maximal lifespan in the Ames (Prop1^df/df^) and Snell (*Pit1^dw^*) dwarf mice,^1^, ^2^ with parallel retardation of age‐related changes in multiple organs and tissues.^3^, ^4^ It seems likely that the lower levels of plasma GH are largely responsible for this decline in the rate of aging, because similar lifespan extension and retardation of multiple age‐dependent changes are seen in mice with disruption of the GH receptor (GHR‐KO),^5^ and because lifespan extension is also seen in mice lacking Ghrh and Ghrhr, which stimulate GH production in the pituitary. Most diseases that can lead to death, including multiple forms of neoplasia, are postponed in all three of these slow‐aging mice.^1^, ^6^ Age‐related decline in cognitive function is also postponed in both Ames and GHR‐KO mice,^7^, ^8^ possibly as a reflection of elevated production of GH within the brain, with concomitant increases in brain cell mitosis.^9^, ^10^

Transient exposure of very young (eg 2 weeks old) Ames dwarf mice to injections of GH, injected at least daily for a period of 2 weeks, can lead to permanent changes of high relevance to the control of aging and late‐life disease. Lifespan of these GH‐treated Ames mice, for example, reverts to that seen in non‐mutant littermate controls.^11^, ^12^ Skin‐derived fibroblasts from Ames, Snell, and GHRKO mice are resistant to multiple forms of cellular injury,^13^, ^14^ but this stress‐resistance phenotype is abrogated in fibroblasts derived from adult Ames mice that had been treated with early life GH injections,^11^ consistent with the finding that mutations that extend lifespan in *Caenorhabditis elegans* typically also lead to resistance to multiple forms of lethal injury,^15^ and to the finding of stress resistance in cells cultured from long‐lived species of rodents, bats,^16^, ^17^ and birds.^18^ Age‐dependent increases in inflammatory status of hypothalamic glial populations, thought to reflect alterations in control of NFkB activation,^19^ are diminished in aging Ames dwarf mice, and this anti‐aging effect is itself blocked when Ames dwarf mice are exposed to transient early‐life GH treatment.^20^ The window of opportunity over which lifelong alterations in anti‐aging pathways can be molded by GH exposure may be quite small, in that GH treatments started at 4 weeks of age do not seem to reduce lifespan either in Snell^21^ or Ames (Bartke et al., unpublished data) mice. Thus, the extent and pace of late life deficits and decline appears to be modulated, to a major extent, by GH effects on one or more unknown cell types if GH levels are modulated within 2 to 4 weeks of birth.

We have recently reported changes in status of both adipocytes and fat‐associated macrophage populations in Snell and GHR‐KO mice.^22^ Levels of the uncoupling protein uncoupling protein 1 (UCP1), a key element in thermogenesis and conversion of fuels to heat,^23^ are elevated in both brown adipose tissue (BAT) and three white adipose tissue (WAT) depots of Snell and GHR‐KO mice, including both subcutaneous (inguinal) and intra‐abdominal (perigonadal and mesenchymal) depots. Adipocytes within the WAT depots also change their size and internal organization with increases in cytoplasm and numbers of mitochondria, a phenomenon referred to as "beige" fat conversion.^24^, ^25^ The macrophage pool within the WAT depots of these long‐lived mutant mice also changes, with an increase in the anti‐inflammatory M2 population and corresponding decrease in the pro‐inflammatory M1 macrophages.^26^, ^27^ Surprisingly, these changes do not appear to reflect actions of GH on the adipocytes themselves, because they are not seen in mice with adipocyte‐specific deletion of GHR. Nor do they reflect effects of IGF‐1, produced by the liver under GH stimulation, because the changes in the fat do not appear in mice with liver‐specific GHR disruption. Disruption of GHR in skeletal muscle, however, does induce nearly all of the changes in adipocyte and fat‐association macrophage subpopulations, implying that the changes in fat are caused by some GH‐regulated myokine acting on fat tissue. A plausible candidate for this role is irisin, whose production by muscle is thought to be increased by exercise.^28^, ^29^ We have reported that irisin is indeed elevated in plasma of Snell and GHRKO mice, consistent with a possible role in modulation of adipose tissues, and further shown an increase, in muscle, of fibronectin type III domain‐containing protein 5 (FNDC5), the protein from which irisin is cleaved and released into the circulation.^22^

A more recent study evaluated control of brain cell biology in Snell and GHRKO mice. Glycosylphosphatidylinositol specific phospholipase D1 (GPLD1),^30^ a plasma protein produced by liver and other tissues including brain, muscle, kidney, and immune cells, cleaves GPI‐anchored proteins from cell membranes, and is increased by exercise in mice and in humans^31^ Elevation of GPLD1 has been shown to improve brain activity in aged mice.^31^ We have reported (Li et al., submitted) that GPLD1 protein is increased in liver and plasma of young adult Snell and GHRKO mice, and that two markers of neuronal maintenance (brain‐derived neurotrophic factor, BNDF) and neurogenesis (doublecortin, DCX) are increased in the hippocampus of both kinds of mice. Interestingly, liver production of GPLD1 is controlled not by transcription, but by selective translation of mRNAs eligible for cap‐independent translation (CIT), a post‐transcriptional pathway that we have shown in other contexts is characteristic of tissues from Snell and GHRKO mice, as well as from mice treated with Rapamycin^32^, ^33^ and other drugs that extend mouse lifespan.^32^ These data suggest a model in which elevated CIT promotes GPLD1 production by liver and/or other tissues, which in turn promotes neurogenesis and, possibly, retention of cognitive skills in aging Ames and GHR‐KO mice.

The experiments reported here address a closely related set of questions: do Ames dwarf mice show the same set of fat, muscle, and brain changes seen in Snell and GHR‐KO mice, and, more importantly, are these physiological changes modulated by the same transient, early‐life, manipulations of GH levels that are known to regulate cell stress, hypothalamic inflammation, and lifespan in the Ames model?

## 2 METHODS

Animals and GH intervention. As previously reported,^12^ 2 week‐old male Ames dwarf (DF) mice were given porcine GH (3 μg/g bw, twice/day) or saline (as vehicle) via s.c. injection for 6 weeks. Heterozygous siblings (df/+) of Ames dwarf mice, which are phenotypically indistinguishable from wild‐type (WT), were used as controls. The mice were maintained on a 12‐h light‐dark cycle with ad libitum access to food (Rodent NIH31 Open Formula Auto with 18% protein and 4% fat, ZEIGLER) and water. Mice were euthanized for tissue collection at 15–18 months of age. Animal protocols were approved by the Animal Care and Use Committee of Southern Illinois University.

Tissue protein extraction and Western blotting. Homogenates were prepared from six tissues (brown fat, inguinal fat, perigonadal fat, liver, skeletal muscle, and hippocampus) in IP buffer (50 mM Tris–HCl pH 7.5, 150 mM NaCl, 1% NP‐40, 5 mM EGTA, 5 mM EDTA, 20 mM NaF, 25 mM β‐glycerophosphate, 0.1 mM sodium vanadate, 1 mM PMSF) supplemented with protease and phosphatase inhibitors (Complete Mini, Roche Diagnostic Corporation; phosphatase inhibitor cocktail, Sigma). Protein concentration was measured using the Bradford method (Protein Assay, Bio‐Rad). 40 µg of protein were resolved by SDS‐PAGE and transferred to PVDF membranes (Millipore). Membranes were blocked with 5% milk in TBS‐T for 1 h and incubated with primary antibodies overnight at 4°C in 5% BSA in TBS‐T (Tubulin 1:4000 and others 1:1000). Proteins were detected by the ECL method and quantified by scanning densitometry. The antibodies used are listed in Table 1.

**TABLE 1:** Source of antibodies

Antibody	Source	Cat. no.
GPLD1	Abcam	210753
BDNF	Abcam	108318
Doublecortin	Abcam	18723
UCP1	Abcam	10983
Arg1	Abcam	124917
iNOS	Abcam	178945
FNDC5	Abcam	174833
Goat anti‐rabbit IgG H&L (HRP)	Abcam	205718
actin	Abcam	9644

Abbreviations: FNDC5, fibronectin type III domain‐containing protein 5; GPLD1, glycosylphosphatidylinositol specific phospholipase D1; UCP1, uncoupling protein 1.

Statistical analysis. For each endpoint, we conducted a one‐way anova, comparing four groups of mice: GH‐treated DF, GH‐treated WT, saline‐treated DF, and saline‐treated WT. A Tukey post‐hoc test, which evaluates all six pair‐wise contrasts and corrects for multiple comparisons, was used to evaluate the two contrasts of particular interest, that is, the difference between DF and WT mice not exposed to GH, and the difference between saline‐treated and GH‐treated DF mice. The effect of genotype is reported as the ratio between DF divided by WT mice (saline treated), so a number higher than 1 represents an increase in DF mice. The effect of GH is reported as the ratio between GH‐treated and saline‐treated DF mice, so a number higher than 1 represents an increase in GH‐treated mice. Protein quantification data normalized to β‐actin and expressed as fold change compared with WT control (defined as 1.0). Data are shown by dotplots where each symbol represents a different mouse. In the graphics, (*) indicates adjusted *p* < .05, and (**) indicates adjusted *p* < .01.

## 3 RESULTS

**FIGURE 1:**
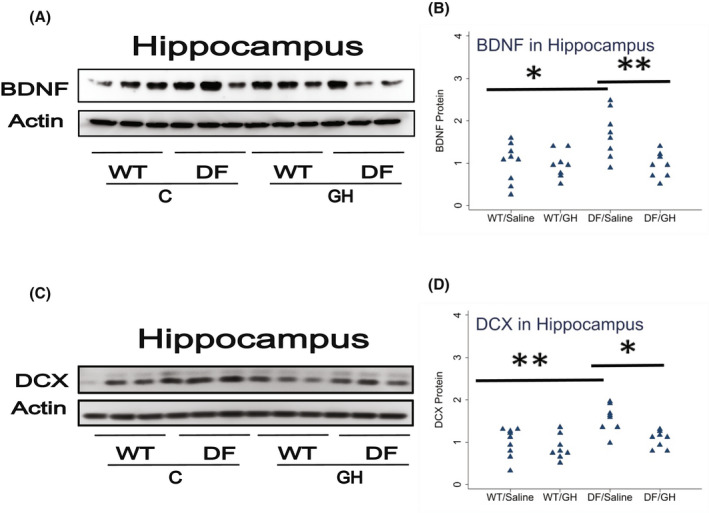
Effect of early life growth hormone (GH) intervention on level of BDNF and Doublecortin (DCX) in hippocampus of Ames dwarf mice. (A,C) Cell lysate was prepared from hippocampus of 15–18 months old wild‐type (WT) littermate control male mice and long‐lived Ames dwarf male mice (DF) with or without GH treatment. Protein levels of BDNF (Panel A) or DCX (Panel C) were then measured by western blotting. Representative gel images are shown. (B,D) Protein quantification data normalized to β‐actin and expressed as fold change compared with WT control (defined as 1.0). *N* = 8 or 9 mice for each group. *p < .05 and **p < .01 by Tukey's post‐hoc test after one‐way anova.

We have recently demonstrated increases in BDNF and DCX in the hippocampus of Snell dwarf and GHRKO mice at age 5–6 months. To see if these proteins are also elevated in hippocampus of Ames dwarf mice, and to extend our data to older mice, we evaluated tissues from Ames dwarf (DF) animals as well as their WT nonmutant controls, with results shown in Figure 1. Half of these mice had been injected with GH twice daily from 2 to 8 weeks of age, and the other half injected with saline as a control. In mice injected with saline, BDNF levels were 1.7‐fold higher in DF mice (*p* = .01), and DCX levels were 1.6‐fold higher (*p* = .002). In mice with the Ames genotype, early‐life GH injection led to a decline, at 20 months of age, in both proteins: BDNF was 56% of that in saline‐injected DF mice (*p* = .009), and DCX was 68% of control levels (*p* = .01). GH did not alter these proteins in the non‐mutant WT control mice. Thus, proteins associated with neurogenesis and maintenance of neuronal function were elevated in Ames DF mice, as they are in young adult Snell and GHRKO mice, a genetic effect reversible by transient early‐life GH exposure. Table 2 summarizes the effect (Ames vs. WT and GH‐Ames vs. Saline‐Ames) for this and the other comparisons presented in this paper.

**TABLE 2:** Collection of statistical results

Tissue	Protein	Ames gene effect (no GH)	GH effect (in Ames mice)
BAT	UCP1	1.89, *p* < .0001	0.66, *p* = .0002
BAT	iNOS	0.72, *p* = .008	1.23, *p* = .21
BAT	ARG1	2.2, *p* < .0001	0.66, *p* = .01
BAT	GPLD1	1.93, *p* < .0002	0.65, *p* = .007
Inguinal fat	UCP1	1.79, *p* = .001	0.60, *p* = .005
Inguinal fat	iNOS	0.60, *p* = .18	1.67, *p* = .19
Inguinal fat	ARG1	1.27, *p* = .49	0.51, *p* = .02
Perigonadal fat	UCP1	1.94, *p* < .0001	0.49, *p* < .0001
Perigonadal fat	iNOS	0.75, *p* < .07	1.15, *p* = .66
Perigonadal fat	ARG1	1.74, *p* < .0007	0.40, *p* < .0001
Hippocampus	BDNF	1.68, *p* = .01	0.56, *p* = .009
Hippocampus	DCX	1.57, *p* = .002	0.68, *p* = .01
Hippocampus	FNDC5	1.70, *p* = .04	0.56, *p* = .03
Hippocampus	GPLD1	1.15, *p* = .51	1.03, *p* = .99
Muscle	FNDC5	1.65, *p* = .0001	0.55, *p* < .0001
Liver	GPLD1	2.20, *p* < .0001	0.48, *p* < .0001

Abbreviations: BAT, brown adipose tissue; GPLD1, glycosylphosphatidylinositol specific phospholipase D1; FNDC5, fibronectin type III domain‐containing protein 5; UCP1, uncoupling protein 1.

**FIGURE 2:**
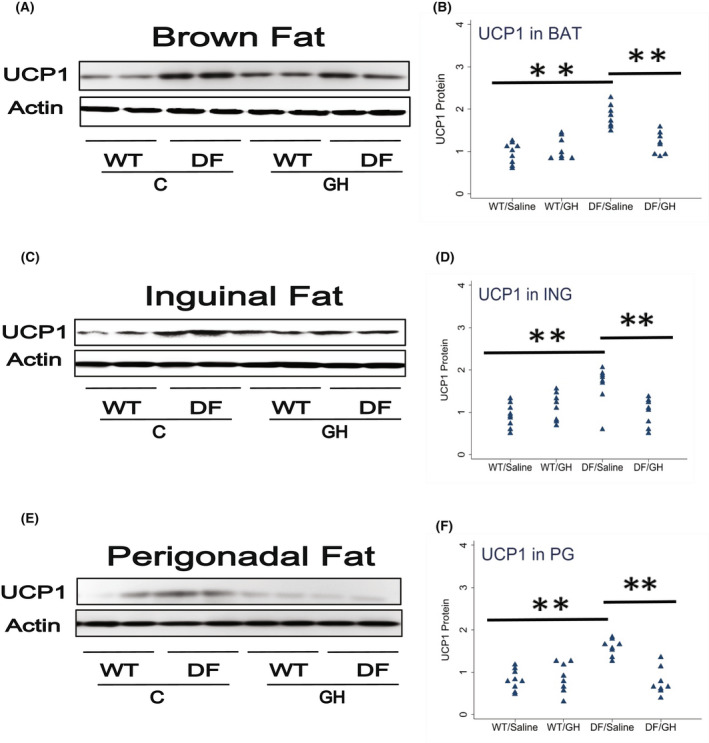
Effect of early life growth hormone intervention on level of uncoupling protein 1 (UCP1) in adipose tissue of Ames dwarf mice. (A) Cell lysate was prepared from brown adipose tissue (Panel A), inguinal fat (Panel C), or perigonadal fat (Panel E) of wild‐type (WT) and DF mice 15–18 months old and evaluated for UCP1 protein by western blotting. Representative gel images are shown. (Panels B,D,F) Protein quantification data normalized to β‐actin and expressed as fold change compared with WT control (defined as 1.0). *N* = 8 or 9 mice for each group. *p < .05 and **p < .01 by Tukey's post‐hoc test after one‐way anova.

Snell dwarf and GHRKO mice also show elevated levels of UCP1 in BAT and WAT, accompanied by other indices of conversion of adipocytes to the beige state, a phenomenon known as "browning" of white fat, in both subcutaneous and intra‐abdominal WAT depots. Figure 2 shows UCP1 levels in BAT, inguinal WAT, and perigonadal WAT in Ames mice with and without early life GH treatment. Ames DF mice have elevation of UCP1 in all three fat depots, with increases of 89%, 79%, and 94% respectively. Early‐life GH injection reduces UCP1 levels to 65%, 60%, and 49%, respectively, returning these levels to those seen in age‐matched WT mice. Each of these effects is significant by Tukey's test compared to the respective controls (see Table 2 for *p*‐values). GH injections did not significantly alter the levels of UCP1 in WT mice. Thus the increase in UCP1 seen in Snell and GHRKO mice is also seen in older Ames mice, and is prevented by early exposure to GH.

**FIGURE 3:**
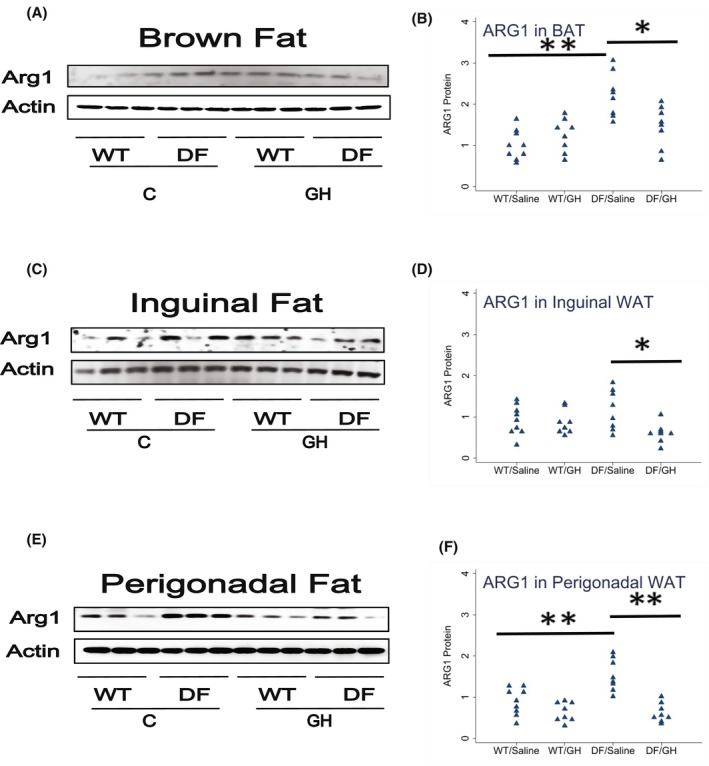
Effect of early life growth hormone intervention on level of Arg1 in adipose tissue of Ames dwarf mice. (A,C,E) Representative gel images. Cell lysate was prepared from brown adipose tissue, inguinal white adipose tissue (WAT), or perigonadal WAT and evaluated for levels of the M2 macrophage marker Arg1. (B,D,F) Protein quantification data normalized to β‐actin and expressed as fold change compared with wild‐type control (defined as 1.0). *N* = 8 or 9 mice for each group. *p < .05 and **p < .01 by Tukey's post‐hoc test after one‐way anova.

Macrophage subpopulations within BAT and WAT depots are also altered in Snell and GHRKO, with increases in ARG1, a marker of the anti‐inflammatory M2 macrophage subset, and declines in iNOS, a marker of the pro‐inflammatory M1 population. Figure 3 presents data on ARG1 in BAT and in inguinal (ING) and perigonadal (PG) white fat of the Ames DF mice. In BAT, ARG1 was increased by a factor of 2.2 in DF mice (*p* < .0001), and early GH treatment reduced ARG1 to a level of 66% compared to saline‐injected DF mice (*p* = .01). Similar effects were seen in perigonadal fat: a 74% increase in ARG1, significant at *p* = .0007, and reduction by GH treatment to levels 40% of those in saline‐treated DF mice. In inguinal fat, ARG1 was 27% higher in Ames than in WT mice, but this difference was not statistically significant (*p* = .5). GH exposure, however, diminished ARG1 levels in inguinal fat (effect size of 51%, *p* = .02). Thus, levels of the M2 macrophage subpopulation are, like UCP1, modulated by early‐life GH exposure in both subcutaneous and intra‐abdominal fat, although elevation of M2 cells by the Ames mutation seems to be stronger in the perigonadal depot.

**FIGURE 4:**
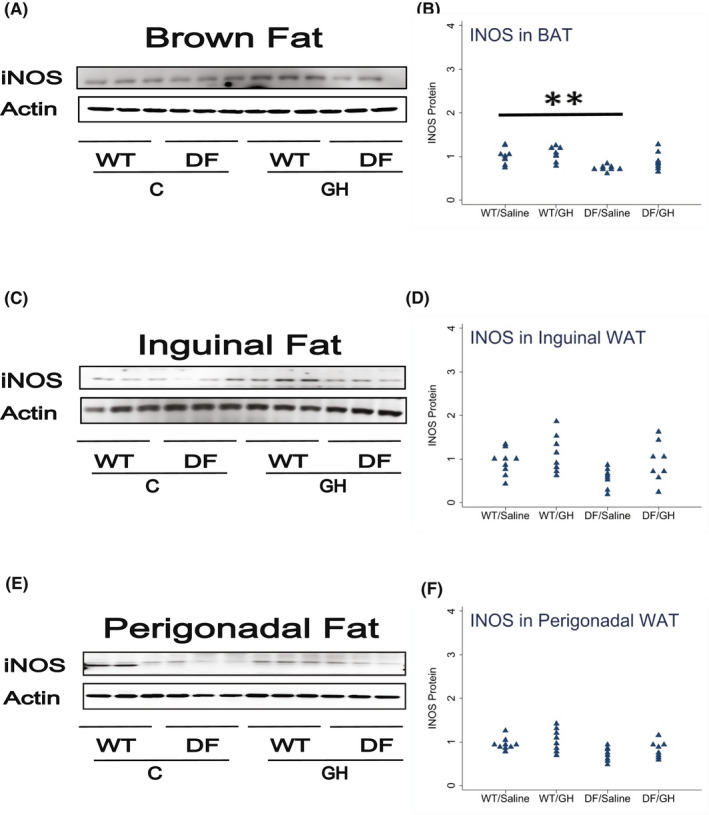
Effect of early life growth hormone intervention on level of iNOS in adipose tissue of Ames dwarf mice. (A,C,E) Representative gel images. Cell lysate was prepared from brown adipose tissue, inguinal white adipose tissue (WAT), or perigonadal WAT, as indicated, and evaluated for levels iNOS protein, a marker of M1 macrophages. (B,D,F) Protein quantification data normalized to β‐actin and expressed as fold change compared with wild‐type control (defined as 1.0). *N* = 8 or 9 mice for each group. *p < .05 and **p < .01 by Tukey's post‐hoc test after one‐way anova.

We also measured iNOS, a marker of M1 cells, in BAT, ING, and PG fat depots. iNOS was diminished in all three depots to varying extents (60%–75% of WT levels), but these did not reach statistical significance. Similarly, early‐life GH exposure led to increases in iNOS levels in all three depots (range: 15%–67%), but these differences did not reach statistical significance (Figure 4).

**FIGURE 5:**
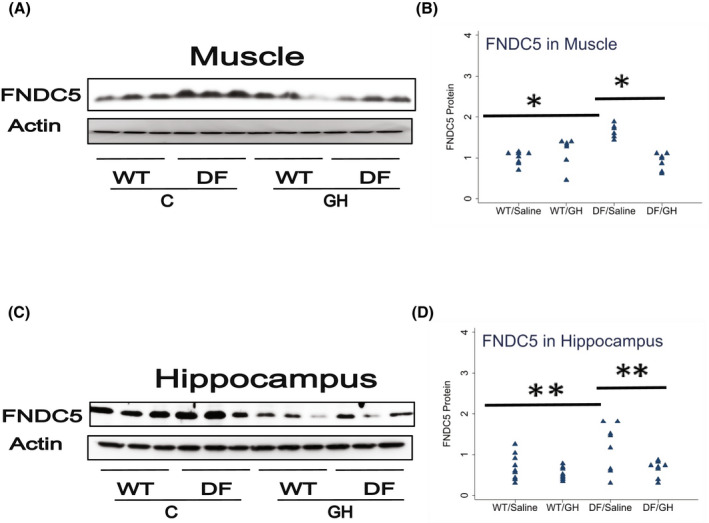
Effect of early life growth hormone intervention on level of fibronectin type III domain‐containing protein 5 (FNDC5) in muscle and hippocampus of Ames dwarf mice. (A,C) Representative gel images. Cell lysate was prepared from muscle or hippocampus, as indicated, and evaluated for levels of FNDC5 protein. (B,D) Protein quantification data normalized to β‐actin and expressed as fold change compared with wild‐type control (defined as 1.0). *N* = 7 or 8 mice for each group. *p < .05 and **p < .01 by Tukey's post‐hoc test after one‐way anova.

We next evaluated the levels of FNDC5, a precursor of the myokine irisin, in skeletal muscle of the Ames mice. As shown in Figure 5, muscle of Ames mice had 65% higher FNDC5 levels than littermate controls (*p* = .0001), and GH injections led to a decline of 55% below that seen in saline‐injected DF mice (*p* < .0001). Because we had previously noted an increase in FNDC5 in brain of other long‐lived mutant mice (Li et al., unpublished), we also measured FNDC5 in the hippocampus of Ames mice, and found a 70% increase in Ames mice compared to littermates (*p* = .04), and a reduction to 56% in the GH‐injected Ames mice (*p* = .03). Thus, modulation of early‐life GH levels appears to control late‐life FNDC5 levels in both muscle and hippocampus.

**FIGURE 6:**
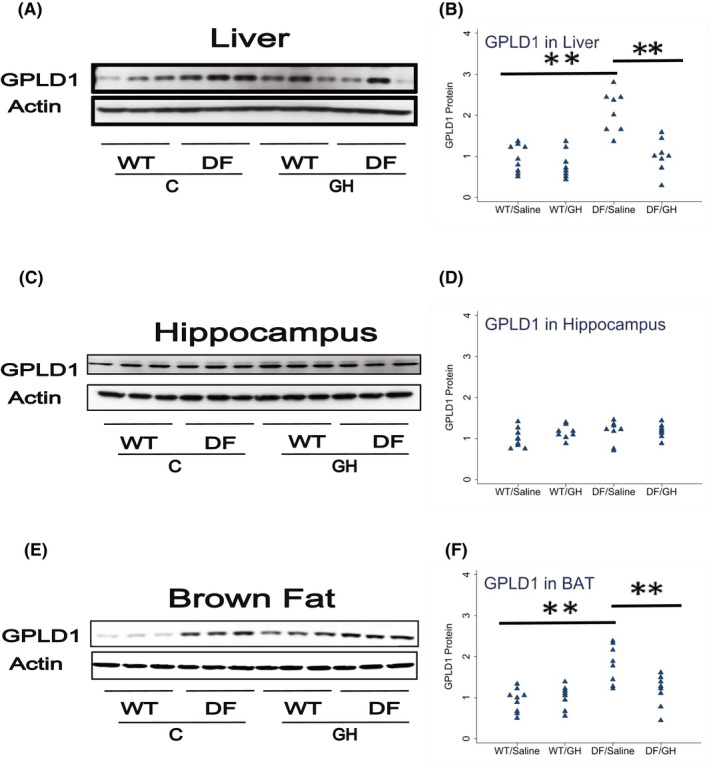
Effect of early life growth hormone intervention on level of glycosylphosphatidylinositol specific phospholipase D1 (GPLD1) in liver, hippocampus, and brown adipose tissue (BAT) in Ames dwarf mice. (A,C,E) Representative gel images. Cell lysate was prepared from liver, hippocampus, or BAT, as indicated, and evaluated for levels GPLD1 protein. (B,D,F) Protein quantification data normalized to β‐actin and expressed as fold change compared with wild‐type control (defined as 1.0). *N* = 8 or 9 mice for each group. *p < .05 and **p < .01 by Tukey's post‐hoc test after one‐way anova.

**FIGURE 7:**
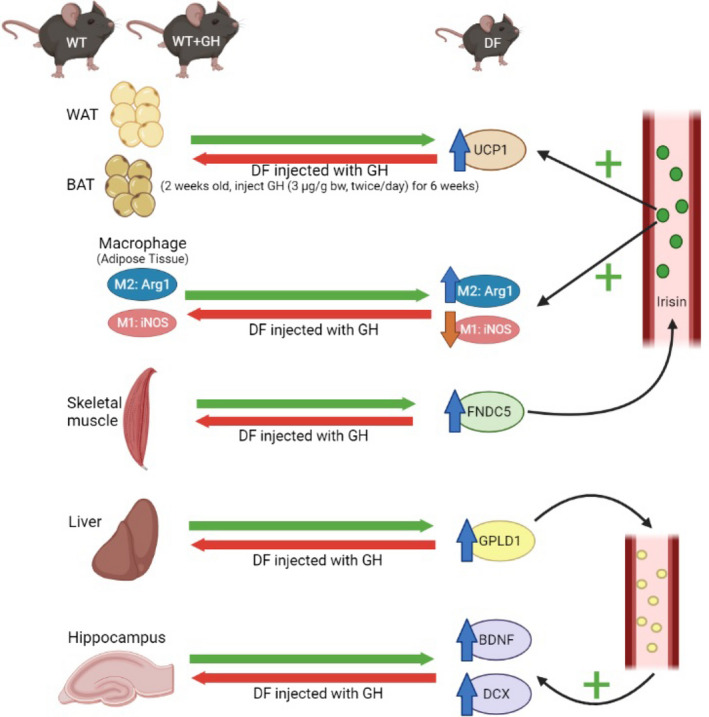
Diagram illustrating the model suggested and supported by the evidence in this paper. Young adult Ames and control mice differ in multiple traits previously seen in slow‐aging Snell dwarf and GHRKO mice, including properties of white adipose tissue, brown adipose tissue, fat‐associated macrophage subsets, liver, and hippocampus. Treatment of juvenile Ames mice with growth hormone (GH) injections for several weeks reverts all of these tissue phenotypes to that of the control animals, suggesting that all these traits can be changed permanently depending on GH levels prior to puberty.

Lastly, we measured GPLD1 protein in BAT, liver, and hippocampus. We have previously shown increased GPLD1 levels in liver and plasma of Snell and GHRKO mice, and shown that these were regulated by the post‐transcriptional process of CIT. Though peripheral GPLD1 levels have been shown^31^ to regulate BDNF and DCX in the brain, and although GPLD1 is produced in the CNS, we had found no change in Snell or GHRKO mice of GPLD1 in the brain itself. In the Ames system, we found a 2.2‐fold increase in GPLD1 protein in liver of DF mice, and reduction by early GH exposure to a level 48% of control, each significant at *p* < .0001 (Figure 6). Similarly, GPLD1 levels in BAT were increased 1.9‐fold in DF mice, with reduction to 65% by GH exposure. Due to restrictions on sample availability, we were not able to evaluate GPLD1 levels in plasma, but we suspect that elevation of GPLD1 production by liver, BAT, and possibly other tissues would have led to higher plasma GPLD1 concentrations. Consistent with our data on Snell and GHRKO mice, we noted no significant change in GPLD1 protein levels in DF hippocampus, and no effect of early GH exposure (Figure 6). Figure 7 provides a diagrammatic summary of the major findings.

## 4 DISCUSSION

### 4.1 Early life GH levels lead to long‐lasting changes in brain, fat, liver, macrophage subsets, and muscle

Our previous report^22^ documented multiple changes in two varieties of long‐lived mice, Snell dwarf, and GHRKO, including increased levels of UCP1 in WAT and BAT, increases in the ratio of M2 to M1 macrophages in fat depots, and increases in the irisin precursor protein FNDC5 in skeletal muscle. These changes were seen in young adult mice, and in each case were opposite in direction to known effects of aging in non‐mutant mice. Follow‐up studies (Li et al., submitted) have shown an increase in plasma and liver levels of GPLD1, and in hippocampal levels of BDNX and DCX, consistent with other reports^31^ that these markers of CNS neurogenesis are increased in response to plasma GPLD1. New data in this report extend this work in several ways. First, we see that most of these alterations are seen in Ames dwarf, another GH‐deficient mutant with extended longevity and delayed aging. Second, we find that the changes noted in young adult Snell and GHRKO mice are apparent in mice 16–18 months of age. Lastly, and most importantly, we find that these effects can be prevented by exposure of DF mice to injected GH for a short period, that is, from 2 to 8 weeks of age. Transient GH exposure has previously been shown to reduce the lifespan benefit of the DF mutation,^12^ to eliminate the stress resistance profile of cell lines derived from skin of adult DR mice,^11^ and to block the protective effects of the DF mutation on several aspects of inflammation in the hypothalamus of aged mice.^20^ Initiation of GH exposure at 4 weeks of age apparently does not reverse the lifespan benefit seen in Snell dwarf mice,^21^ nor in Ames DF mice (Bartke et al., unpublished results), suggesting that susceptibility to GH signals relevant to life‐long patterns of aging and late‐life timing of diseases may be greatest in a short period within the first few weeks of post‐natal life. However, studies involving deletion of the Ghr gene at 6 weeks or 6 months of age indicate that GH signaling during adult life has a role in determination of longevity in female mice.^34^, ^35^ Interestingly, in these studies longevity of males was not affected by disrupting GH signaling during adult life.

Glycosylphosphatidylinositol specific phospholipase D1 and indices of brain cell status: The availability of archived tissues from an experiment in which Ames DF mice were exposed to GH from 2 to 8 weeks of age allowed us to test a range of endpoints that are likely to play a role in delayed aging in long‐lived mutant mice with diminished GH production or responsiveness. Adult neurogenesis occurs throughout life in the dentate gyrus of mammalian hippocampus in mammals.^36^ With aging, adult hippocampal neurogenesis declines in mammals.^37^, ^38^ Ames mice, like GHRKO mice,^7^ retain relatively high levels of cognitive performance at ages where controls begin to lose these abilities.^39^ Our recent work shows, in the Snell and GHRKO models, elevation of GPLD1 in liver and plasma (Li and Miller, submitted). GPLD1 has been shown to increase indices of neurogenesis (DCX and BDNF) in mouse brain, and we have found elevations of both proteins in brains of Snell, GHRKO, and now also Ames mice, consistent with a model in which GPLD1, produced by liver, fat, or other tissues, leads to improved neurogenesis, turnover of brain stem cells,^9^ and, perhaps, maintenance of cognitive function in older animals. In this context, it is noteworthy that elevated production of GPLD1 seems to reflect selective translation of GPLD1 mRNA by CIT, a mode of post‐transcriptional control characteristic of Snell, GHRKO, and Pappa‐KO mice.^33^ Our current data show elevation of GPLD1 production by both liver and BAT of DF mice, and it is possible that elevation of plasma GPLD1 in each of these long‐lived models reflects production of this protein by more than one of these and other sources. Although hippocampus does contain detectable GPLD1 protein, its levels do not significantly differ between Ames and WT mice, and are not modified by early‐life GH treatment, suggesting still‐undefined CNS pathways for detecting and responding to peripheral, that is, plasma, levels of GPLD1.

### 4.2 Uncoupling protein 1 and thermal homeostasis

Uncoupling protein 1 (UCP1), a mitochondrial membrane protein critical to adaptive thermogenesis, is particularly high in brown adipocytes.^40^ Cells with similar morphological features (such as smaller size, increased cytoplasm and higher mitochondrial mass), and higher expression of UCP1, can also be found in WAT depots in mice and humans^41^, ^42^; these cells are referred to as "beige" fat cells, and their production is referred to as "browning" of WAT. Aging is characterized by an increase in WAT adiposity, and relatively decreased amounts of BAT depots and UCP1 levels within brown adipocytes, as well as a decline in the ability to produce beige adipocytes in WAT depots.^42^ During aging, UCP1 is reduced in BAT and WAT in both mice and humans.^43^, ^44^ The amount of BAT tissue, and level of UCP1 in BAT, are both increased in Ames DF mice,^45^ consistent with the idea that higher BAT thermogenic activity might contribute to longevity and health maintenance in DF mice. We have also reported higher levels of UCP1 in BAT, as well as in subcutaneous and intra‐abdominal fat of Snell and GHRKO mice.^22^ Other indices of thermogenic activity, including PPARγ, DIO2, and ADRβ3, are also elevated in these mice. There are also morphologic changes: adipocytes in BAT of normal mice contain small, round, lipid vacuoles that appear as unstained areas in sections stained with hematoxylin and eosin, while in contrast BAT adipocytes are smaller, with more cell nuclei per microscopic field, and have reduced droplet size. Our new data show elevated UCP1 in both BAT and WAT depots of middle‐aged Ames DF mice, and also show that this trait can be inhibited by brief early‐life GH exposure in each depot tested. We have not tested thermogenesis per se in these mice, but we would predict improved thermogenic ability, and perhaps also resistance to the harmful effects of high fat or high calorie diets, in these mice. There is considerable evidence that UCP1 mediated uncoupling of the mitochondrial electron transport chain can be beneficial for health related metabolic traits including beta oxidation of fatty acids and glucose homeostasis, and can promote healthy aging and extended longevity. Consistent with this concept, BAT function is enhanced in Ames dwarf mice and surgical removal of interscapular BAT has greater impact on oxygen consumption and expression of genes related to thermogenesis and lipid metabolism in these long‐lived mutants than in their WT siblings.^34^ Moreover, Ames dwarf mice are resistant to the detrimental impact of high fat diet on insulin sensitivity, adiponectin levels, and energy expenditure.^46^

Genetic and pharmacological interventions that elevate longevity in mice are often associated with resistance to age‐related increases in intra‐abdominal fat, with relative preservation of subcutaneous WAT depots.^47^ Similarly, surgical removal of intra‐abdominal fat can lead to a lifespan increase in rats that is not seen after surgical removal of a similar amount of subcutaneous WAT.^48^ These observations suggest that the balance between subcutaneous and intra‐abdominal fat may be particularly relevant to aging and health maintenance. In this context, it is noteworthy that browning and UCP1 elevation in Snell, Ames, and GHRKO mice appears to affect both intra‐abdominal as well as subcutaneous WAT. Beiging of WAT in response to various stimuli is much more pronounced in the inguinal (subcutaneous) than in the epididymal (visceral) WAT depots, and in some cases is observed only at the former site.^49^, ^50^

### 4.3 Macrophage polarization

Aging leads to increasing levels of low‐grade inflammation, which are hypothesized to contribute to multiple late‐life diseases.^51^, ^52^, ^53^ Changes with age in adipose tissue macrophages (ATMs) may also play a role in systemic or local adipose tissue inflammation.^54^ The ratio of pro‐inflammatory M1 ATMs to anti‐inflammatory M2 ATMs increases significantly with age in mice.^54^ Our previous study documented increases in M2 cells, with parallel decreases in M1 macrophages, in BAT and two WAT depots of 6 months old Snell and GHRKO mice,^22^ using Arg1 protein levels as a marker for M2 cells and iNOS as a marker for M1 macrophages. Our current data set reflects work done using a different mutation, on a different (and shorter‐lived) background stock, raised in a different vivarium (Southern Illinois University rather than the University of Michigan), and focuses on mice that are much older than those used in our prior studies. Some of the findings seen in young Snell dwarf mice were also noted in the older Ames DF, including increases in M2 and decreases in M1 in BAT, increases in Arg1 M2 cells in PG fat, and a marginal (*p* = .07) decline in M1 cells in PG fat. Changes seen for Arg1 and for iNOS cells in inguinal fat were in the expected direction, but did not reach statistical significance. Early life GH exposure opposed each of these shifts in M1/M2 polarization in BAT, and the changes in Arg1 M2 cells in both WAT depots, but produced changes in the expected direction for iNOS cells in the WAT depots that did not reach significant levels (see Table 1 for summary). We do not know at present if changes in fat depot M1 and M2 cell populations are caused by alterations, such as browning, of the adipocyte populations, or by direct or indirect effects of GH itself on macrophage subset differentiation. Similarly, we do not know whether changes in the adipocytes are themselves secondary to alteration in macrophage or other stromal elements in the fat depots of these mice. But our previous study showed that deletion of GH receptor in adipocytes did not recapitulate changes in UCP1, Arg1, or iNOS, arguing against direct GH effects on the fat cells per se. Similarly, liver‐specific GH deletion did not reproduce the effects of global GH deficit, implying that declines in GH‐triggered hepatic IGF1 production were not sufficient to alter adipocytes or M1/M2 ratios. Most of the changes seen in GHRKO and Snell fat depots were, however, seen in mice where GHR had been depleted in skeletal muscle.

### 4.4 Fibronectin type III domain‐containing protein 5 in muscle and brain

Bostrom et al^28^ demonstrated that exercise stimulates muscle to produce FNDC5, a membrane protein that is cleaved and secreted as a newly identified hormone, irisin. Irisin acts on white adipose cells in culture and in vivo to stimulate UCP1 expression and a broad program of changes characteristic of conversion of white to brown fat cells. FNDC5 expression can be induced in mouse hippocampus by exercise, and it in turn activates BDNF and other neuroprotective genes.^55^

Our new data show increases in FNDC5 protein in skeletal muscle of Ames dwarf mice, similar to our findings in younger adult Snell and GHRKO animals.^22^ Lack of available sample prevented us from measuring irisin, the cleavage product of FNDC5, in serum from these mice, although the higher levels of FNDC5 imply that irisin is likely to have been elevated in these Ames DF mice, and the browning and UCP1 elevation in the fat depots is entirely consistent with models linking these fat traits to muscle FNDC5/irisin production. Elevations of muscle FNDC5 were ablated in mice subjected to early life GH exposure (decline to 55% of saline‐treated Ames mice, *p* < .0001), suggesting that homeostatic set‐points for this muscle protein, too, were permanently altered by GH levels before and shortly after weaning. Interestingly, we also noted an increase in FNDC5 in hippocampus of Ames DF mice, which was in turn blocked by early exposure to GH. FNDC5 is also higher in brain tissue of Snell and GHRKO mice (Li and Miller, unpublished data). Thus, our data suggest that modulation of early‐life GH levels can control late‐life FNDC5 levels in both muscle and hippocampus. We speculate that higher FNDC5 in muscle, brain, or perhaps other tissues can, perhaps via irisin signals, influence beige differentiation of adipocytes and perhaps also macrophage polarization. It is unclear whether FNDC5, or its cleavage products, can act either locally in the brain or after secretion to the peripheral circulation, to influence inflammatory and metabolic control pathways that extend lifespan and delay late‐life illness in Ames, Snell, and GHRKO mice. The role of GPLD1, FNDC5, and other paracrine and endocrine factors as mediators of early‐life set‐points of aging rate offer multiple opportunities for further investigation. Studies to evaluate levels and activities of these factors in mice in which lifespan has been increased by rapamycin, acarbose, 17α‐estradiol, canagliflozin, and other anti‐aging drugs are now under way.

## AUTHOR CONTRIBUTIONS

Richard A. Miller, Andrzej Bartke, and Xinna Li conceived the experiment. Yimin Fang and Andrzej Bartke prepared the materials from Ames dwarf mice. Xinna Li, Madaline McPherson, and Mary Hager produced the data. Xinna Li did the statistical analysis and prepared the figures. Xinna Li and Richard A. Miller wrote the manuscript.

## DISCLOSURES

The authors have stated explicitly that there are no conflicts of interest in connection with this article.

## Data Availability

All raw images, densitometric data, and statistical calculations are available from the authors (XL, RAM) on request.
